# Raman Spectroscopy for the Competition of Hydrogen Bonds in Ternary (H_2_O–THF–DMSO) Aqueous Solutions

**DOI:** 10.3390/molecules24203666

**Published:** 2019-10-11

**Authors:** Shiliang Liu, Mingzhe Zhang, Baokun Huang, Nannan Wu, Shunli Ouyang

**Affiliations:** 1 Key Laboratory of Integrated Exploitation of Bayan Obo Multi-Metal Resources, Inner Mongolia University of Science and Technology, Baotou 014010, China; 13224726207@163.com (S.L.); imust2016023081@163.com (M.Z.); 2 School of Energy and Environment, Inner Mongolia University of Science and Technology, Baotou 014010, China; 3 Electronic Engineering School, Huaihai Institute of Technology, Lianyungang 222000, China; huang_baokun@163.com; 4 College of Science, Inner Mongolia University of Science and Technology, Baotou 014010, China

**Keywords:** Raman spectroscopy, hydrogen bond, binary aqueous solution, ternary aqueous solution, fully hydrogen-bonded water

## Abstract

The effects of hydrogen bonds on the molecular structure of water-tetrahydrofuran (H_2_O–THF), water-dimethyl sulfoxide (H_2_O–DMSO), and water-tetrahydrofuran-dimethyl sulfoxide (H_2_O–THF–DMSO) in binary aqueous solutions and ternary aqueous solutions were studied using Raman spectroscopy. The results indicate that in the binary aqueous solution, the addition of THF and DMSO will generate hydrogen bonds with water molecules, resulting in changes in the peak positions of S=O bonds and C–O bonds. Compared with the binary aqueous solutions, the hydrogen bonds between DMSO and THF, and the hydrogen bonds between DMSO and H_2_O in the ternary aqueous solutions are competitive, and the hydrogen bond competition is susceptible to water content. In addition, the formation of hydrogen bonds will destroy the fully hydrogen-bonded water and make it change to the partially hydrogen-bonded water. By fitting the spectra into the three Gaussian components assigned to water molecules with different hydrogen bonding (HB) environments, these spectral features are interpreted by a mechanism that H_2_O in different solution systems has equal types of water molecules with similar HB degrees-fully hydrogen-bonded H_2_O (FHW) and partially hydrogen-bonded H_2_O (PHW). The ratio of the intensity transition from FHW to PHW is determined based on Gaussian fitting. Therefore, the variation of hydrogen bond competition can be supplemented by the intensity ratio of PHW/FHW ((I_C2_ + I_C3_)/I_C1_). This study provides an experimental basis for enriching the hydrogen bonding theory of multivariate aqueous solution systems.

## 1. Introduction

Water, as a simple and peculiar substance in nature, has been widely used in many fields, and has become a hot scientific topic of widespread concern [1,2,3,4,5]. There are many mysteries to it, for example, to date, it is the only substance found to have an abnormal density. Unlike other substances whose density reaches its maximum in the solid state, it expands during the liquid–solid phase transition and reaches a maximum at 276.984 K [5]. Russo et al. [6] found that the physical properties of water near 276.984 K were related to the transformation of different hydrogen bonding modes in water by means of molecular mechanics simulation.

Hydrogen bonding, as the basis of water structure, is an important microscopic effect in aqueous systems [7,8,9]. As a bridge between bonding molecules, it affects the microscopic heterogeneity and micro-structure of the solutions. Thus, the hydrogen bonding being a key factor for many anomalous properties of water with the change of micro structure [1,10,11,12]. Taking the binary mixture of water and organic liquids as an example, the increased content of the organic liquid typically destroys the three-dimensional water–hydrogen bond, thereby changing the macroscopic properties of the solution [13]. For instance, in a binary solution (DMSO–H2O), mixing DMSO with water can effectively prevent the formation of hydrogen bonds between the S=O bond and the O–H bond, which restricts the formation of the ice tetrahedral structure when the temperature drops below 273 K [14]. Therefore, DMSO–H_2_O binary mixture solution with appropriate proportion can be used as excellent antifreeze material. The effect of hydrogen bonds on the surface tension of a binary mixture (acetone-water) was studied using Raman spectroscopy. The experimental results indicate that the surface tension of the solution will gradually weaken with the increase of hydrogen bonds between acetone. The basic experimental data of the spectral analysis confirms the relationship between the micro-structure and macroscopic properties of aqueous solutions [15]. The stability, activity, melting point and density of the substance change significantly with the formation of hydrogen bonds. The study of hydrogen bond structure is helpful for us to better construct the relationship between the micro-structure and macroscopic properties of aqueous solutions. However, there is no complete system for the law of hydrogen bond interaction in binary, ternary and multivariate aqueous solution systems. In binary mixtures of water and organic liquids, it is found that the increase of organic liquids usually destroys the three-dimensional water–hydrogen bond [13]. Compared with ternary aqueous solutions, binary aqueous solutions are only hydrogen bonds between water molecules and water molecules, organic solution molecules and water molecules, while ternary aqueous solutions may involve hydrogen bond interactions between different organic molecules, which needs further analysis.

NMR spectroscopy [16,17] and vibrational spectroscopies (IR and Raman [18,19,20,21,22,23]), are useful for investigating hydrogen bonding interactions in solutions. The OH stretch region is particularly informative because OH stretch wave numbers are very sensitive to local molecular environments [24,25]. However, this band is notably broadened and highly asymmetric so that it has not been fully understood. Many authors deconvoluted the OH stretch band into several components (usually three or more), that are assigned to water molecules engaged in different HB environments. For example, Li et al. [26] fitted the OH stretch band with five Gaussian components assigned to fully or partly hydrogen-bonded water molecules. In a study on hydrogen bonding in different solution systems, we observed the change of the intensity ratio of PHW/FHW in the OH stretch region in terms of (I_C2_ + I_C3_)/I_C1_. On the other hand, the intensity ratio of PHW/FHW is also useful for analyzing the competition between DMSO and HO for THF in ternary aqueous systems.

In this paper, Raman spectroscopy is used to study the interaction between different substances in ternary aqueous solutions (H2O–THF–DMSO). It is found that not only the hydrogen bond between THF, DMSO and H2O molecules, but also the Raman peak position of C–O and S=O, and the hydrogen bond between THF and DMSO can cause the shift of the Raman peak position.

## 2. Results and Discussion

### 2.1. Raman Spectroscopy of Binary Aqueous Solution

**Figure 1:**
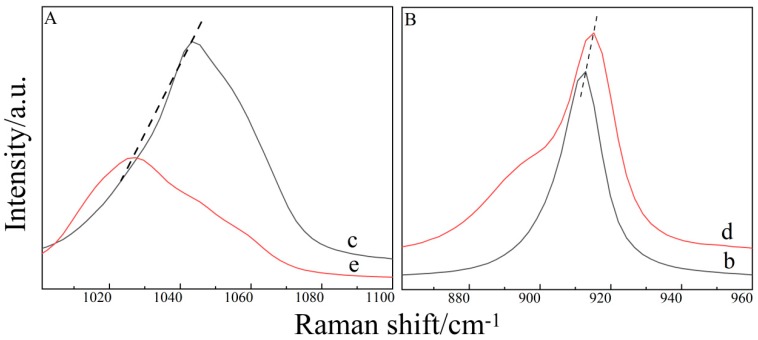
Raman spectra of binary aqueous solutions. (**A**) pure DMSO (Sample c) vs. H_2_O–DMSO solution (Sample e). (**B**) pure THF (Sample b) vs. H_2_O–THF solution (Sample d).

Figure 1A presents the Raman spectra of the S=O bond vibration peak in a pure DMSO (Sample c) and a H_2_O–DMSO binary aqueous solution (Sample e) at normal temperature. It shows that the S=O bond moves to a low wave number after adding water to DMSO, which indicates that the vibration frequency of the S=O bond decreases. The relationship between vibration frequency and vibration force constant (κ) is as follows [27]:

(1) $$\nu =\frac{1}{2\pi c}\sqrt{\frac{\kappa }{\mu }}$$

where *c* stands for velocity of light, µ stands for the reduced mass of diatomic molecules. The decrease of frequency indicates that the vibration force constant decreases, and the vibration force constant is related to the density of the shared electron pairs electronic cloud. When the electron cloud density shifts between two atoms, the vibration force constant increases and vice versa [27]. The original electron cloud is biased towards the side of the S atom with a larger atomic mass. After the O atom and the H atom form a hydrogen bond, the electron cloud is slightly distant from the O atom and deviates further from the center of the two atoms. Therefore, the vibration force constant is reduced. According to the relationship between the vibration force constant and the vibration frequency, the vibration frequency of the S=O bond should be reduced, which is consistent with the experimental observation. The addition of water to DMSO produces a hydrogen bond between the two, causing the S=O bond to be elongated and the vibration frequency to move to a low wave number [28].

**Figure 2:**
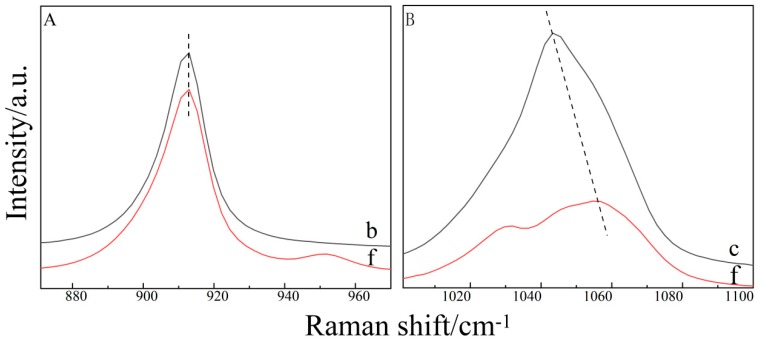
The Raman shift of C–O and S=O. (**A**) THF and THF–DMSO. (**B**) DMSO and THF–DMSO.

Figure 1B shows the Raman spectra of C–O in neat THF (Sample b) and the H_2_O–THF binary solution (Sample d) [11,29,30,31,32]. As can be seen from Figure 2, water is added to the THF, and the peak position of the C–O bond shifts to the high wave number. A hydrogen bond is formed between the O atom of THF and the H atom of the H_2_O molecule, which shortens the C–O bond and increases the vibration frequency, leading to the blue shift of the C–O bond.

### 2.2. Raman Spectroscopy of the Binary THF–DMSO Solution

To further explore the interaction between THF and DMSO in a binary mixed solution, we performed a more detailed analysis of their experimental results.

**Figure 3:**
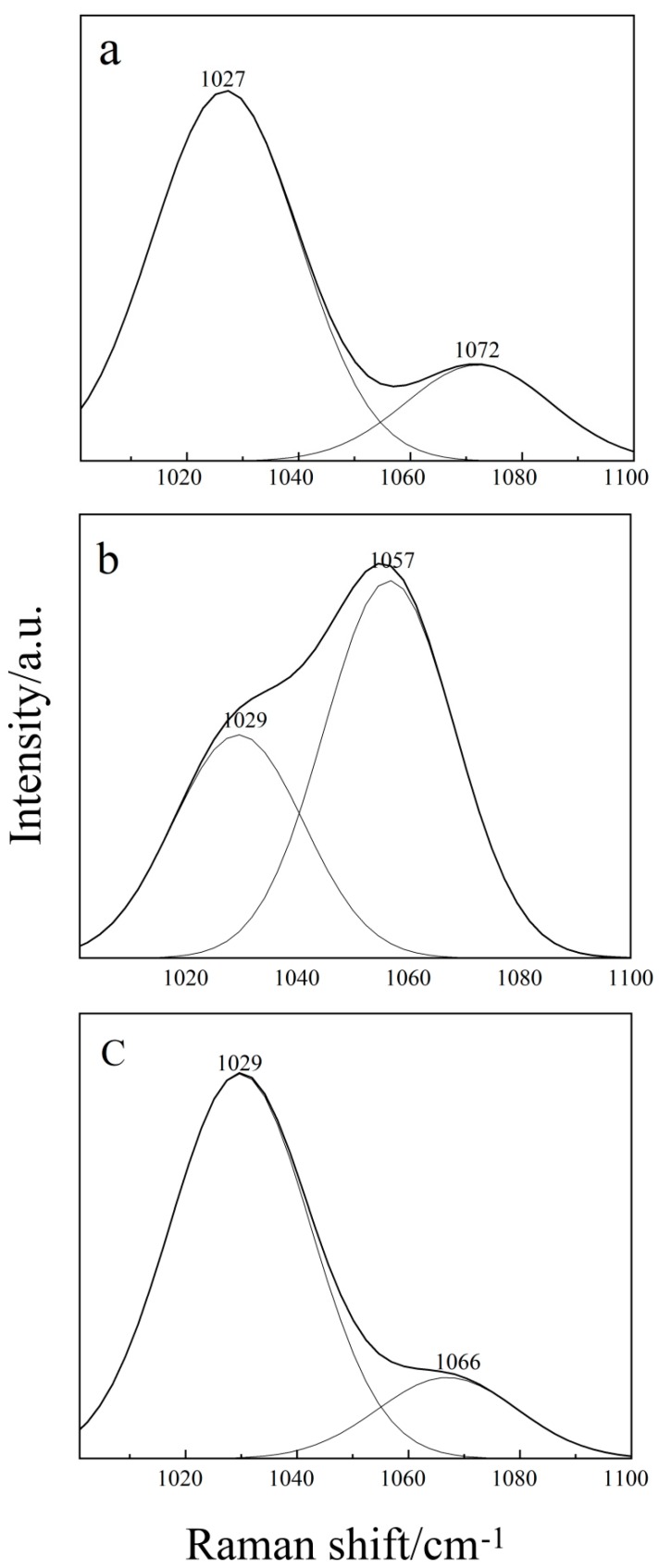
Raman shift vs. hydrogen bonding between different solutions. (**a**) pure THF, (**b**) THF–DMSO solution, and (**c**) H_2_O-THF.

By comparing the Raman spectra of C–O in the pure THF solution and the THF–DMSO binary mixed solution (Figure 2A), we found that, with the addition of DMSO, the peak shape and position of the C–O bond remained unchanged, indicating that binary mixed solution did not contribute to the C–O bond. By observing the change of the S=O bond in the binary mixture solution (Figure 2B), we found that its Raman peak position and peak pattern of THF–DMSO binary solution changed noticeably in the range of 1000–1100 cm^−1^ wave number, and was divided into two peaks. The results are shown in Figure 3b.

The Raman spectra of pure THF recorded in the region 1000–1100 cm^−1^ show two peaks, one intense peak at 1027 cm^−1^ and another peak which is less intense at 1072 cm^−1^ (as shown in Figure 3a). These two Raman bands may be assigned as the combination bands of different ν(C–C) stretching and ν(C–H) bending modes [32]. In the binary H_2_O–THF aqueous solution (as shown in Figure 3c), although the C–C and C–H peaks shift due to their interaction, the intensity peak is still strongest and the second is weak. While in THF-DMSO binary solution, adding DMSO not only makes the peak blue shift at 1027 cm^−1^ and the red shift at 1072 cm^−1^, but it also reverses the contrast of the peak intensity completely. Compared with Figure 3a,c, the peak at 1057 cm^−1^ is stronger than that at 1027 cm^−1^. Obviously, this is due to the hydrogen bonding between THF and DMSO. It shows that the H atom from the C–H bending mode of the neat THF, may interact with the O atom from S=O of the DMSO, which not only lengthens the C–H bond and leads to a red shift, but also shortens the S=O bond and leads to a blue shift. Finally, the superposition of C–H and S=O results in the enhancement of Raman peaks at 1057 cm^−1^. Furthermore, the blue shift of C–C is due to the change of the C–H bond, which results in the overall configuration of the C–C band.

Another explanation for the blue shift of C–C and S=O may be offered in terms of increasing the corresponding force constant, while the red shifts of the C–H band will be caused by the decrease of the force constants, which may be a consequence of the transfer of an electronic charge cloud from the C–H bond to the C–C ring and S=O bond. This is similar to the results discussed by other researchers [33]. A shorter bond length essentially implies an increase in the force constant too.

### 2.3. Raman Spectroscopy of the Ternary Aqueous Solution

**Figure 4:**
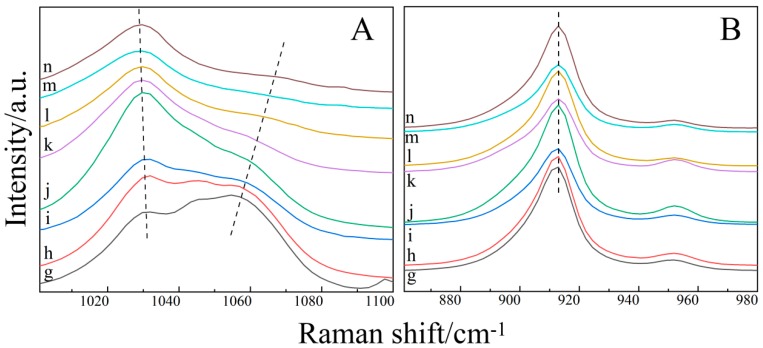
Raman shift of S=O (**A**) and C–O (**B**) vs. water content of the ternary solution.

The Raman shift of S=O and C–O vs. the water content of the ternary solution is further compared in Figure 4. As shown in Figure 4A, the central wave number of C–C displacement hardly migrates lower than the C–H shift by approximately 10 cm^−1^ (from 1060 cm^−1^ to 1070 cm^−1^) as the water content increases from 0.1:1:1 to 2.5:1:1.

From the point of view of the change of the S=O bond wave number of DMSO, in sample g (*n*(H_2_O):*n*(THF):*n*(DMSO) = 0.1:1:1), the Raman spectra in the range of 1000–1100 cm^−1^ are similar to those of sample f (*n*(H_2_O):*n*(THF):*n*(DMSO) = 0:1:1). This indicates that the hydrogen bonding between DMSO and THF is stronger than that between H_2_O and water, when the water content of the ternary aqueous solution is low. The hydrogen bond between DMSO and THF makes the S=O bond blue shift and the C–H bond red shift, which overlap. From sample g to sample n, the peak at 1060 cm^−1^ is gradually weakened and shifted to approximately 10 cm^−1^ in the direction of the high wave number. This is due to the enhanced hydrogen bonding between DMSO and H_2_O with the increase of the water content, and the hydrogen bonding between DMSO and H_2_O will destroy the hydrogen bond between DMSO and THF. From sample i to sample j, the peak strength at 1029 cm^−1^ increased significantly, because the hydrogen bond between H_2_O and DMSO was stronger than that between THF and DMSO. As in the binary H_2_O–DMSO solution (sample e), the S=O bond red shift overlaps with the C–C bond under the hydrogen bond of H_2_O and DMSO.

In addition, the Raman shift of the C–O bond vs. the concentration of the aqueous solution is shown in Figure 4B. It shows that the C–O bonds of THF with different concentrations of ternary aqueous solutions are almost unchanged. This also demonstrates that the hydrogen bond between DMSO and THF is stronger than that between H_2_O and THF at a lower water content. At a higher water content, the hydrogen bond between H_2_O and DMSO is also stronger than that between H_2_O and THF, thus inhibiting the formation of a hydrogen bond between H_2_O and THF. When forming clusters corresponding to a range of different mole fractions, up to four water molecules are added to each DMSO molecule [34]. We boldly speculate that, when the content of water in a ternary aqueous solution is much higher than that in DMSO and THF, the inhibition of a hydrogen bond between DMSO and H_2_O and the formation of a hydrogen bond between THF and H_2_O is greatly weakened. Furthermore, the change of the C–C bond is the same as that in the binary H_2_O–THF (sample d), and the blue shift of the C–C bond occurs slightly.

**Figure 5:**
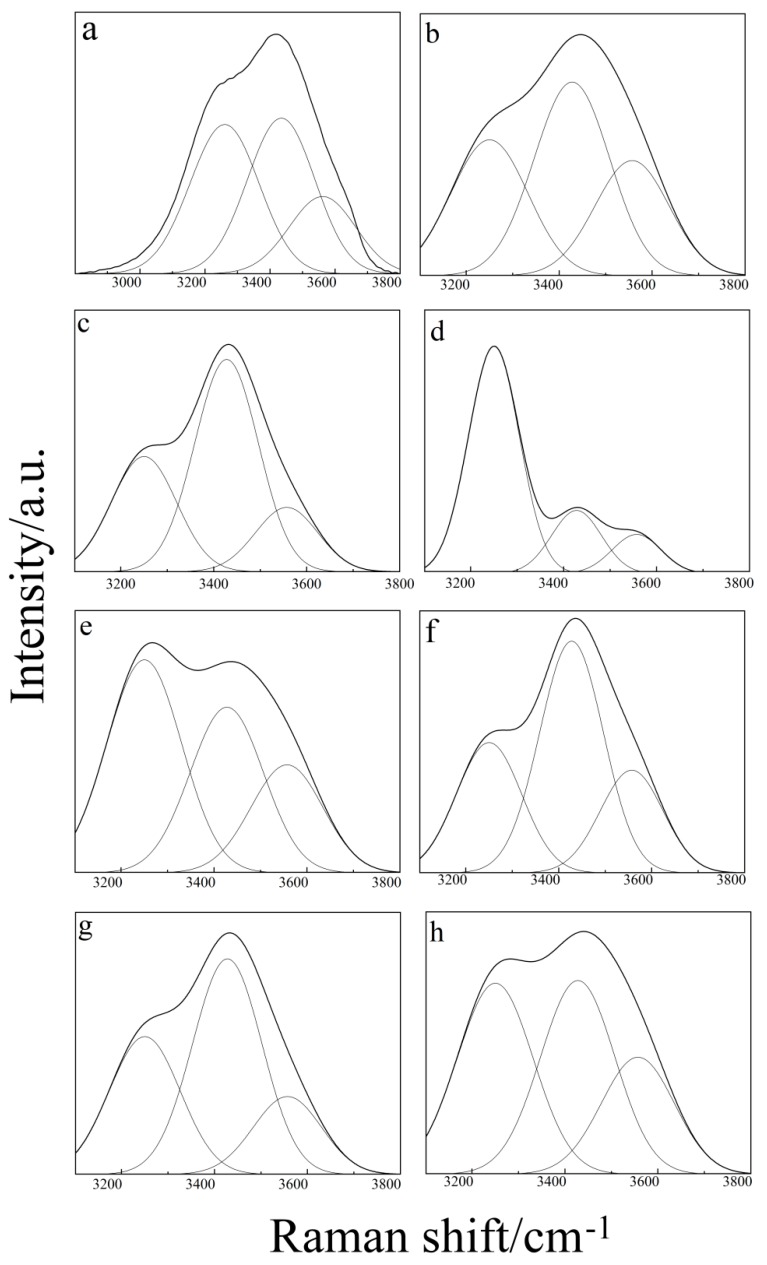
Deconvolution of Raman spectra into three Gaussian components C1, C2 and C3 with the central wave number at 3245, 3420 and 3550 cm^−1^, for (**a**) sample a, (**b**) sample d, (**c**) sample e, (**d**) sample g, (**e**) sample h, (**f**) sample j, (**g**) sample k, and (**h**) sample m. The thicker dashed lines represent the smoothed line of the raw spectral data by the Peak Fit software. The superposed contour is the fitted line by the three Gaussian components.

In order to verify our hypothesis in ternary aqueous solutions, we make a supplementary analysis from the transformation of fully hydrogen-bonded water to partially hydrogen-bonded water and the ratio of intensity. In this paper, peak fit software is used to divide the characteristic peaks of the OH bend. The relative peak intensity of each dividing peak is shown in Figure 5. The Raman spectra of the characteristic peaks of the OH bond are decomposed into three Gaussian components, Pek I, Pek II and PeK III. The components are located at 3245 cm^−1^ (C1), 3420 cm^−1^ (C2) and 3550 cm^−1^ (C3). The former component was assigned to the fully hydrogen-bonded water (FHW), while the second and third were assigned to the partially hydrogen-bonded water (PHW) [20], and their peak intensities are expressed by I_C1_, I_C2_, and I_C3_, respectively.

Changing the concentration of the aqueous solutions, the transition from FHW to PHW can be described by the following relationship:

C1→C2 + C3

Furthermore, the ratio of intensity from FHW to PHW can be described by the following relationship:

(I_C2_ + I_C3_)/I_C1_

The transition from I_C2_ + I_C3_ to I_C1_ of ratio of intensity in Figure 5 is systematically counted. The results are shown in Table 1.

From pure water (sample a) to binary (sample d, sample e) and ternary (sample g, sample h, sample j, sample k, sample m) aqueous solutions, by observing the Raman spectra of Figure 5, and based on the aforementioned analysis and discussion, we propose that pure water, binary and ternary aqueous solutions have equal types of molecules with similar HB degrees (fully hydrogen-bonded, partially hydrogen-bonded) to pure water. However, HB configurations in pure water, binary and ternary aqueous solutions are different. The Raman spectra of binary and ternary aqueous solutions and pure water can both be well-fitted with three Gaussian components, but the rate of PHW relative to FHW (PHW/FHW) is different.

From the pure water to the binary aqueous solution, Figure 5a–c and Table 1 show that the hydrogen bonding between THF and water, DMSO and water can destroy FHW and transform it into PHW. However, the values of (I_C2_ + I_C3_)/I_C1_ for the H_2_O–THF binary aqueous solution is slightly lower than that of the H_2_O–DMSO binary aqueous solution. This fact suggests an easier breakage of the HB structure in the H_2_O–DMSO binary aqueous solution than in the H_2_O–THF binary aqueous solution, which also shows that hydrogen bonding is more easily formed between DMSO and H_2_O.

Furthermore, in the ternary aqueous solution, the rate of PHW relative to FHW (PHW/FHW) differs from that of pure water to the binary aqueous solution (Figure 5d–h) in nearly the same conditions. Table 1 shows an increasing trend of the intensity ratio of (I_C2_ + I_C3_)/I_C1_, when *n*(H2O):*n*(THF):*n*(DMSO) < 1:1:1, but gradually decreasing when *n*(H2O): *n*(THF):*n*(DMSO) > 1:1:1. The reason for this phenomenon is that, as we analyzed earlier, an increasing content of water strengthens the hydrogen bond between DMSO and H_2_O, and competes with the hydrogen bond between DMSO and THF in the case of *n*(H2O):*n*(THF):*n*(DMSO) < 1:1:1. However, in the case of *n*(H2O):*n*(THF):*n*(DMSO) > 1:1:1, a part water and DMSO have hydrogen bonding, while the others exist in the form of pure water. Therefore, the content of FHW increases, and the ratio of intensity from FHW to PHW (I_C2_ + I_C3_/I_C1_) also increases.

## 3. Experimental

### 3.1. Reagents and Instruments

Ultrapure water, HPLC grade, with a resistivity of 18.2 MΩ cm was purchased from J&K Scientific LED (Beijing, China). The DMSO used in the experiment is in purity greater than 99.9%, without further purification before the experiment. The THF used in the experiment was also not further purified before experiment, and its purity is more than 99.9%. The mixture of H_2_O, THF and DMSO was prepared with a certain proportion, and the specific molar ratio parameters are shown in Table 2.

Raman spectroscopy was collected by the Raman spectrum system constructed independently by our laboratory equipped with an Andor Sham-rock SR-500i-C-R type Raman spectrometer (Andor, Belfast, UK), an Andor iDus series water-air cooled CCD (Charge Coupled Device) detector product by the UK Andor company (Belfast, UK), and a 1200 grove/mm grating with a wavelength resolution of approximately 0.05 nm. In the experiment, a 50 times long focus Olympus objective lens (parameter: 50×/0.35, Olympus Corporation, Tokyo, Japan) was used to focus on samples. After adjusting the three-dimensional sample stage height to complete the focal point, the focusing image was collected by a SunTime130 E CMOS color digital camera (Thorlabs, Newton, NJ, USA), excited at the 532 nm line of a semiconductor laser (Cobolt, Stockholm, Sweden) where the output power was ~25 mW. Then, the Raman spectrometer was calibrated with a diamond standard sample. All the signal dates were gathered at room temperature with 5 s exposure time, 2 times accumulation number, 5 s accumulation cycle time, and the scanning range was 0 to 4000 cm^−1.^

### 3.2. Sample Preparation

The reagents used for sample preparation are H_2_O, THF and DMSO. The moles of each component are shown in Table 2. The reagents were fully mixed and tested at room temperature.

## 4. Conclusions

Raman spectroscopy has been used to investigate the effects of hydrogen bonds on the molecular structure of H_2_O–THF, H_2_O–DMSO and H_2_O–THF–DMSO in binary aqueous systems and ternary aqueous systems at normal temperature. The results indicate that because of the formation of hydrogen bonds between H_2_O–THF and H_2_O–DMSO, there are changes in the Raman shifts of S=O and C–O bonds, and this destroys the fully hydrogen-bonded water. In addition, in ternary (H_2_O–THF–DMSO) aqueous systems, a hydrogen bond is formed between DMSO and water, THF and DMSO. As there will be competition between them, this hydrogen bond competition is susceptible to the influence of water content. The Raman spectroscopy clarifies hydrogen bonding in ternary aqueous solutions as follows:

(1) Hydrogen bonding in ternary aqueous solutions occurs between H_2_O–DMSO and THF–DMSO.

(2) When *n*(H_2_O):*n*(THF):*n*(DMSO) < 1:1:1, the increase in water content strengthens the hydrogen bond between DMSO and water, and weakens the hydrogen bond between DMSO and THF.

(3) When *n*(H_2_O):*n*(THF):*n*(DMSO) > 1:1:1, the hydrogen bond competition between DMSO and water will further inhibit the hydrogen bond competition between DMSO and THF, and the remaining water exists in the form of pure water.

## Figures and Tables

**Table 1:** Ratio of intensity (I) sum of C2, C3 (I_C2_ + I_C3_) to intensity the sum of C1 (I_C1_) for H_2_O, binary and ternary aqueous solutions.

Sample	a	d	e	g	h	j	k	m
(I_C2_ + I_C3_)/I_C1_	1.58	2.20	2.40	0.45	1.30	2.55	2.14	1.60

**Table 2:** Molar contents of each sample.

Sample	*n*(H_2_O):*n*(THF):*n*(DMSO)	Sample	*n*(H_2_O):*n*(THF):*n*(DMSO)
a	1:0:0	h	0.3:1:1
b	0:1:0	i	0.5:1:1
c	0:0:1	j	1:1:1
d	1:1:0	k	1.5:1:1
e	1:0:1	l	2:1:1
f	0:1:1	m	2.5:1:1
g	0.1:1:1	n	3:1:1
